# Are 3D Printing Templates an Advantage in Upper Thoracic Pedicle Screw Fixation?

**DOI:** 10.7759/cureus.13989

**Published:** 2021-03-19

**Authors:** Ismail Kaya, İlker Deniz Cingöz, Meryem Cansu Şahin, Murat Atar, Safak Ozyoruk, Murat Sayin, Nurullah Yuceer

**Affiliations:** 1 Neurosurgery, Usak University, Usak, TUR; 2 Medical Physics, Kutahya Health Sciences University, Kutahya, TUR; 3 Neurosurgery, Abdulhamid Han Research and Training Hospital, Istanbul, TUR; 4 Neurosurgery, Private OFM Antalya Hospital, Antalya, TUR; 5 Neurosurgery, Private Saglık Hospital, İzmir, TUR; 6 Neurosurgery, İzmir Katip Çelebi University, İzmir, TUR

**Keywords:** 3d printing, pedicle screw, preoperative plan, upper thoracic trauma

## Abstract

Background

This study aims to compare the clinical results of patients with upper thoracic vertebral fractures treated with pedicle screw and posterior spinal fusion with preoperative surgical planning and 3-dimensional (3D) modeling and patients treated with freehand screws.

Methods

Fifty patients who underwent pedicle screw placement with a diagnosis of upper thoracic fracture between June 2018 and October 2020 were included in our study. Pedicle screws were used in 25 patients (group 1) after the planning was completed with the help of 3D preoperative printing and modeling. Pedicle screws were applied in 25 patients in the control group (group 2) using the freehand technique. Intraoperative bleeding amount, operation time, and correct screw placement data in both groups were recorded.

Results

The operation time was 134 ± 22 minutes for group 1 and 152 ± 38 minutes for group 2. The difference in operation times was found to be statistically significant (p < 0.05). Based on axial and sagittal reconstruction images, the accuracy rate of pedicle screw placement (grades 0 and 1) in group I was 96.6% compared to 83.6% in group II. The minor perforation rate (grade 1, <2 mm) was 5.8% in group I compared to 11.8% in group II. The moderate perforation rate (grade 2, 2-4 mm) was 3.4% in group I compared to 14% in group II. The severe perforation rate (grade 3, >4 mm) was 2.3% in group II; however, misplaced screws were not associated with neurological deficits. The difference in overall accuracy rates between the two groups was significant (p < 0.05).

Conclusions

For 3D models of upper thoracic pedicle screw insertion, guide plates can be produced inexpensively and individually. It provides a new method for the accurate placement of upper thoracic pedicle screws with high accuracy and secure use in screw insertion.

## Introduction

Spinal cord injuries are common in patients with upper thoracic vertebral fractures due to the smaller diameter of the spinal canal compared to the cervical and lumbar regions. Posterior screw fixation is applied in the upper thoracic region and is used in trauma, segmental instability, kyphosis, scoliosis, infection, and tumor treatments [1, 2]. Pedicle screw fixation provides rigid intervertebral fixation but associated with complications, such as artery injury, nerve root damage, and dural damage [3, 4].

Advantages of surgical stabilization in upper thoracic vertebral injuries are correction of sagittal and coronal balance and neurological decompression in kyphotic fractures. Additionally, fixation and fusion prevent hyperkyphosis [5]. Anatomical studies indicate that thoracic pedicle screw insertion in the upper segments is more difficult than that in the lower segment due to the narrower diameter of the pedicles [6]. Moreover, the visualization of the upper thoracic region by X-ray and C-arm fluoroscopy during surgery is limited. This limitation of imaging in the preoperative period increases the risk of screw malposition. A number of studies reveal that inaccuracies in placement using these traditional techniques range from 10% to 50% [7]. In the authors' experience with image guidance in over 1500 cases, several potential pitfalls have been identified that could lead to sub-optimal results when using intraoperative spinal navigation [7].

Currently, 3-dimensional printing technology is used in the preoperative evaluation of patients' anatomy, prosthesis, and implant applications. 3D printers help to increase surgical success by providing a preoperative simulation of surgical approaches [8, 9]. Models created with 3D compression are used in medical and surgical fields, such as cranial surgery, maxillofacial traumas, tissue engineering, chest deformities, and complex spine surgery [8, 10].

Creating patient-specific 3D printing models reduces complications during surgery by providing the surgeon with preoperative surgical planning and application. This study aims to compare the clinical results of patients with upper thoracic vertebral fractures treated with pedicle screw and posterior spinal fusion with preoperative surgical planning and 3D modeling and patients treated with freehand screws.

## Materials and methods

This article was previously posted to the Research Square preprint server on January 07, 2021. Patients diagnosed with upper thoracic fractures between June 2018 and October 2020 were evaluated according to Thoraco‐Lumbar Injury Classification and Severity score (TLICS). Patients were deemed as appropriate candidates for spinal stabilization based on TLICS score of 5 or greater; patients suffering from upper thoracic trauma at T1-T6 segment accompanied with incomplete or complete spinal cord injury, and requiring surgery; patients with senile osteoporotic vertebral fracture and other upper thoracic trauma, not requiring surgery. The final diagnosis was based on the thoracic vertebral fracture and spinal cord injury in thoracic magnetic resonance imaging. Patients who had previously undergone spine surgery, preoperative radiation, chemotherapy, or had recurrent tumors and younger than 18 years were excluded from the study. Fifty patients with pedicle screw implantation were included in this study. Pedicle screws were applied in 25 patients (group I) after planning was completed with the help of 3D preoperative modeling. Pedicle screws were applied in 25 patients in the control group (group II) using the freehand technique. 3D printing was performed in the Kutahya Health Sciences University Research and Application Laboratory.

Digital design and 3D printing

**Figure 1:**
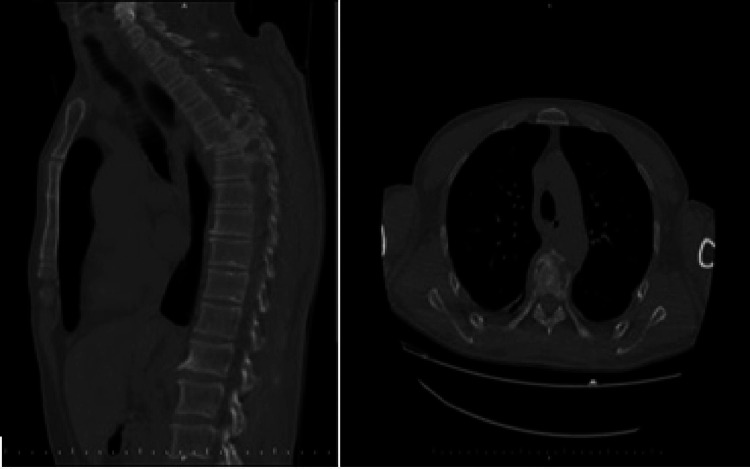
Preoperative CT view of the upper thoracic fracture.

Preoperative computed tomography (CT) images of 25 patients diagnosed with an upper thoracic fracture in the Neurosurgery Clinic were used for 3D models. Preoperative digital imaging and communications in medicine (DICOM) images of each patient were reconstructed using 32-channel computed tomography at a slice thickness of 0.625 mm and a planar resolution of 0.35 mm (Aquilion™ Large Bore CT, Canon Medical Systems, Tustin, USA) (Figure 1).

**Figure 2:**
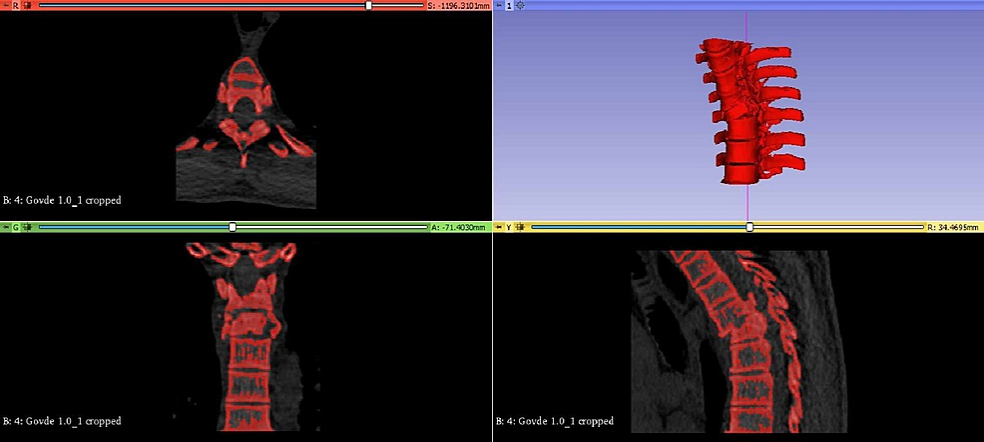
Modeling of the vertebrae in the 3DSlicer program.

CT images containing approximately 450 sections for each model were transferred to the 3D Slicer (version 4.10.1, Boston, MA, USA) program to create a 3D vertebral model. Using this software, the images were used to create 3D models of the vertebral region related to the complex surface treatment method. Whereas only the vertebral model was created from preoperative DICOM images (Figure 2). The templates to be used for pedicle screw placement were modeled in SolidWorks 2015 SP5 software (SolidWorks Corporation, Waltham, MA, USA) according to the measurements obtained from CT images and 3D vertebra models.

**Figure 3:**
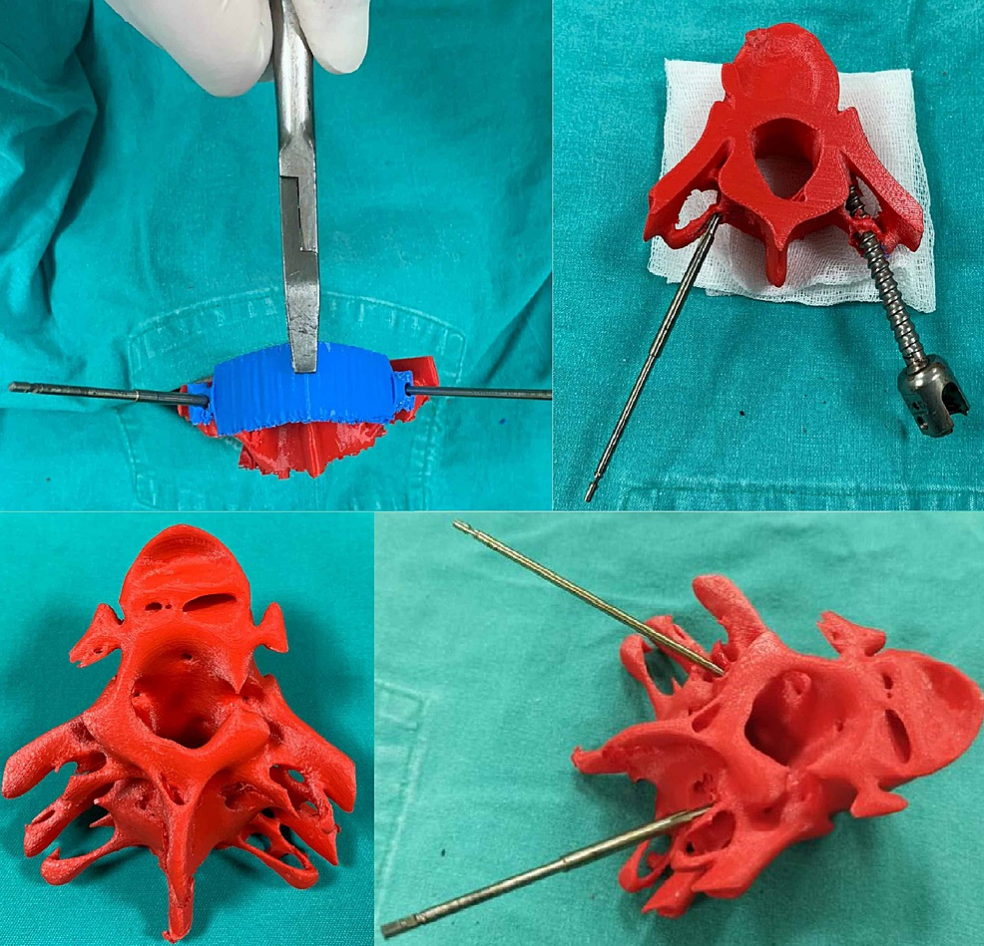
T4-T6 vertebrae prepared for preoperative planning.

3D vertebrae model and template data were saved in stereolithography (STL) format and transferred to Ultimaker Cura (version 4.7.1) (Ultimaker B.V., Utrecht, Netherlands) software. Printing parameters for preoperative vertebral models and templates were prepared in Ultimaker Cura software. The following printing parameters were used for Ultimaker 2 Extended 3D printer and polylactic acid (PLA) in Ultimaker Cura software for the printing of preoperative models: 0.4 mm nozzle diameter, 200°C nozzle temperature, 70°C build plate temperature, and 70% filling rate. Preoperative planning studies were performed on vertebra models and templates by the relevant surgeon (Figure 3).

Operational methods

Patients’ operations were performed by the same surgeons. Preoperative modeling and planning of the patients to be operated on was done with the help of 3D printing and modeling methods. Patient-specific full-scale spine models were available for reference at the time of surgery. Pedicle screws were placed from anatomic regions previously determined by planning and checked with fluoroscopy. In the control group patients, pedicle screws were placed using the freehand technique and under fluoroscopic control.

Evaluation of efficacy

Intraoperative bleeding amount, operation time, and correct screw placement data in both groups were recorded. Intraoperative bleeding was calculated by subtracting the volume of fluid used for flushing from the total fluid volume in the suction bag. The time of insertion of each pedicle screw was recorded.

A control CT scan was performed after the operation. Screw malpositions and violations of the medial and lateral walls of the pedicles were noted. The position of the screws was evaluated according to the Gertzbein classification [11]. In this classification, there are four categories for screw placement: grade 0, screws are completely within the pedicle; grade 1, perforation < 2 mm; grade 2, perforation between 2 and 4 mm; and grade 3, perforation > 4 mm. In the current study, grades 0 and 1 were considered satisfactory, whereas grades 2 and 3 were regarded as perforated.

Statistical analysis

The statistical analysis was performed using SPSS 24.0 (IBM Corp., Armonk, NY, USA) software. Data are presented as the mean ± SD (x±s), and intergroup comparisons were performed with independent-samples t-tests. The enumeration data are expressed as a ratio, and intergroup comparisons were performed with the chi-square test; p = 0.05 was used as the statistical inspection standard.

## Results

Of the 50 patients diagnosed with upper thoracic fractures, 25 were female, and 25 were male. The mean age of these 50 patients was 37.3 ± 5.9 years. No statistically significant difference was found between the groups in terms of age and gender (Table 1).

**Table 1:** Comparative demographic data of both groups. Ϯ Compared with between group 1 and group 2

	Group 1	Group 2	p-value ^Ϯ^
Number of Patients	25	25	-
Sex	14 Male / 11 Female	13 Male / 12 Female	0.184
Age	36.4 ± 6.2	38.1 ± 5.7	0.260

Based on axial and sagittal reconstruction images, the accuracy rate of pedicle screw placement (grades 0 and 1) in group I was 96.6% compared to 83.6% in group II (Table 2). The minor perforation rate (grade 1, <2 mm) was 5.8% in group I compared to 11.8% in group II. The moderate perforation rate (grade 2, 2-4 mm) was 3.4% in group I compared to 14% in group II. The severe perforation rate (grade 3, >4 mm) was 2.3% in group II; however, misplaced screws were not associated with neurological deficits (Table 2). The difference in overall accuracy rates between the two groups was significant (p < 0.05).

**Table 2:** Classification of patients according to Gertzbein scoring. Ϯ Accuracy = (grade 0 + grade 1)/n x 100%

Misplacement (according to Gertzbein’s classification)	Group 1 (n = 174 screws)	Group 2 (n = 171 screws)
Grade 0 (screws are completely within the pedicle)	158 (90.8%)	123 (71.9%)
Grade 1 (screw perforation < 2 mm)	10 (5.8%)	20 (11.8%)
Grade 2 (screw perforation between 2–4 mm)	6 (3.4%)	24 (14%)
Grade 3 (screw perforation > 4 mm)	-	4 (2.3%)
Accuracy Ϯ	96.6%	83.6%

The operation time was 134 ± 22 minutes for group 1 and 152 ± 38 minutes for group 2. The difference in operation times was found to be statistically significant (p < 0.05) (Table 3). The amount of blood loss for group 1 was 962 ± 108 mL. For group 2, it was 992 ± 114 mL. The difference in the amount of blood loss was not statistically significant (p > 0.05) (Table 3).

**Table 3:** Surgical data. Ϯ Compared with between group 1 and group 2

	Group 1 (n = 25)	Group 2 (n = 25)	p-value ^Ϯ^
Operation Time (min)	134 ± 22	152 ± 38	p < 0.05
Blood Loss (mL)	962 ± 108	992 ± 114	p > 0.05

**Figure 4:**
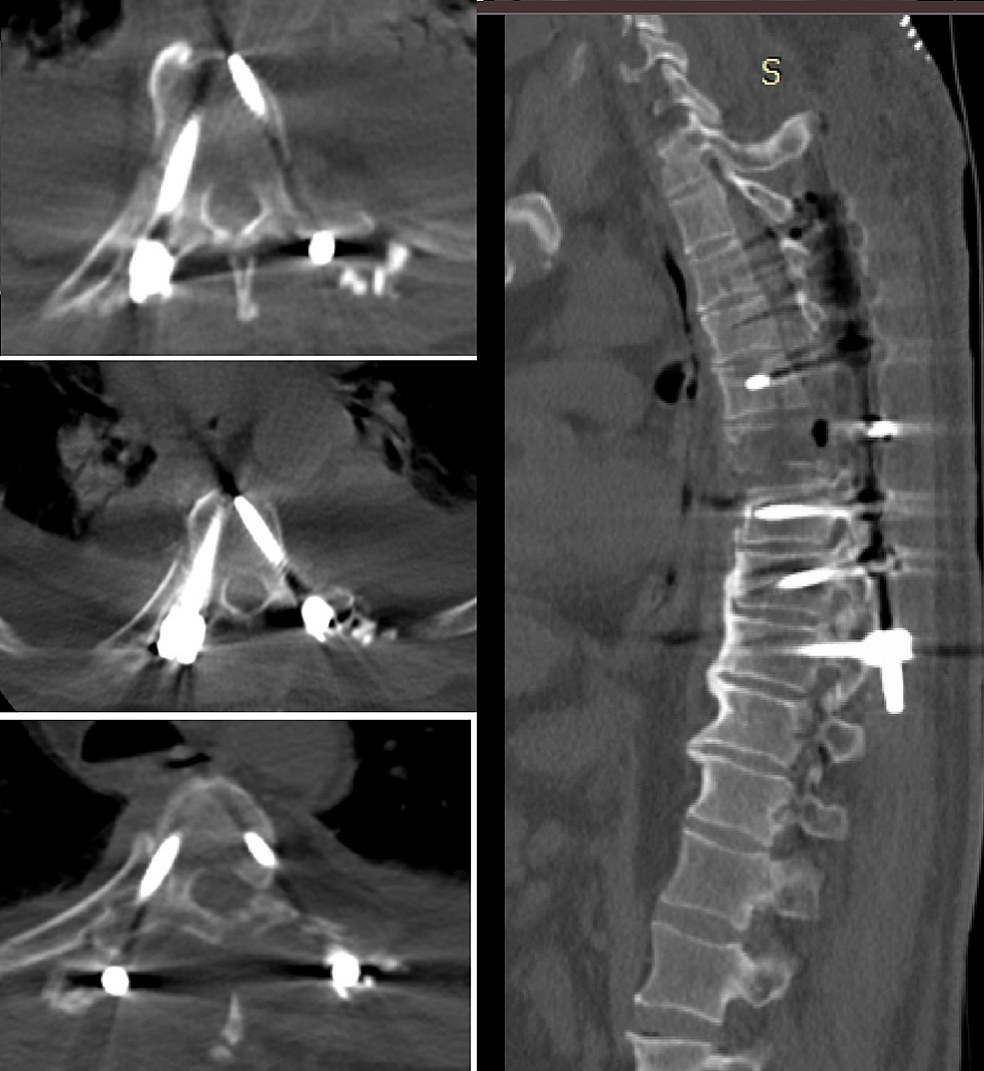
Postoperative CT image of a male patient operated with free hand technique.

**Figure 5:**
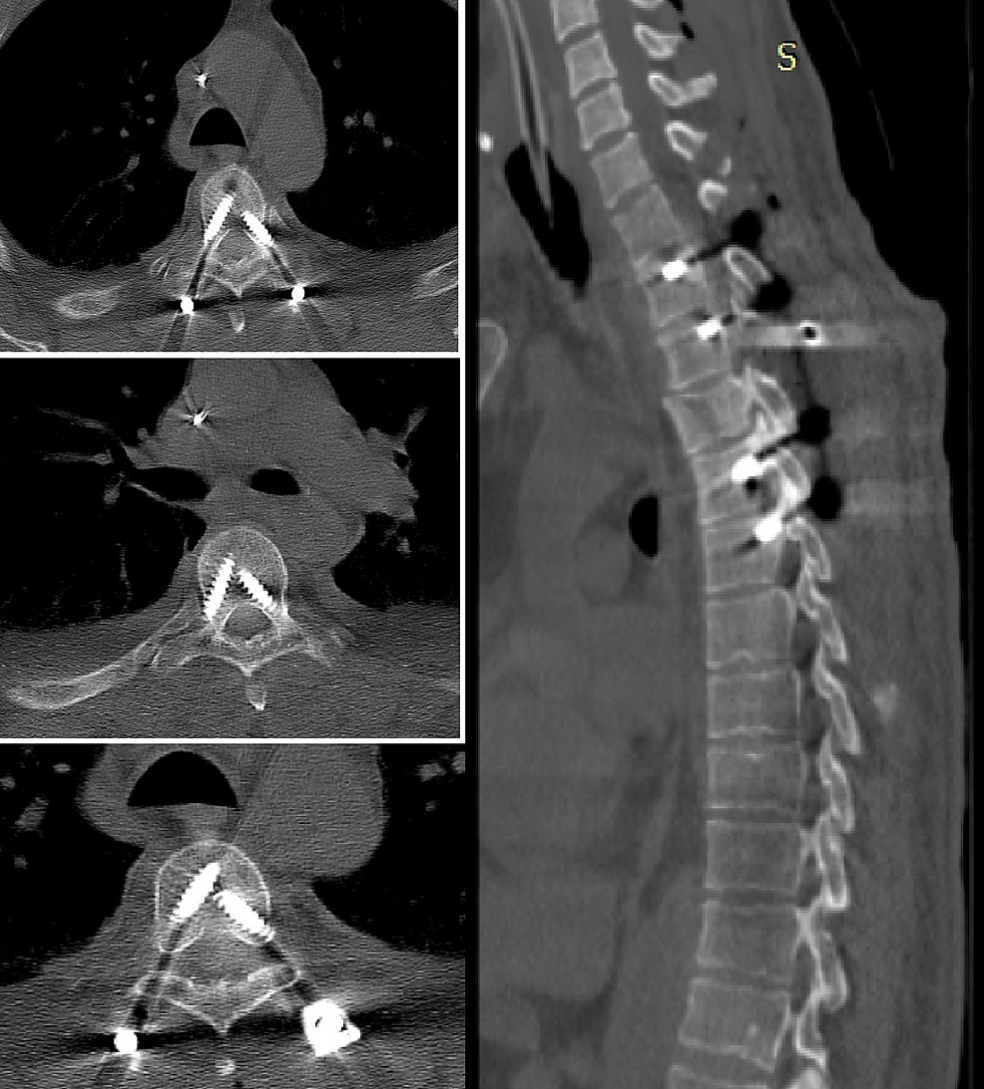
Postoperative CT image of a female patient operated with 3D modeling technique.

**Figure 6:**
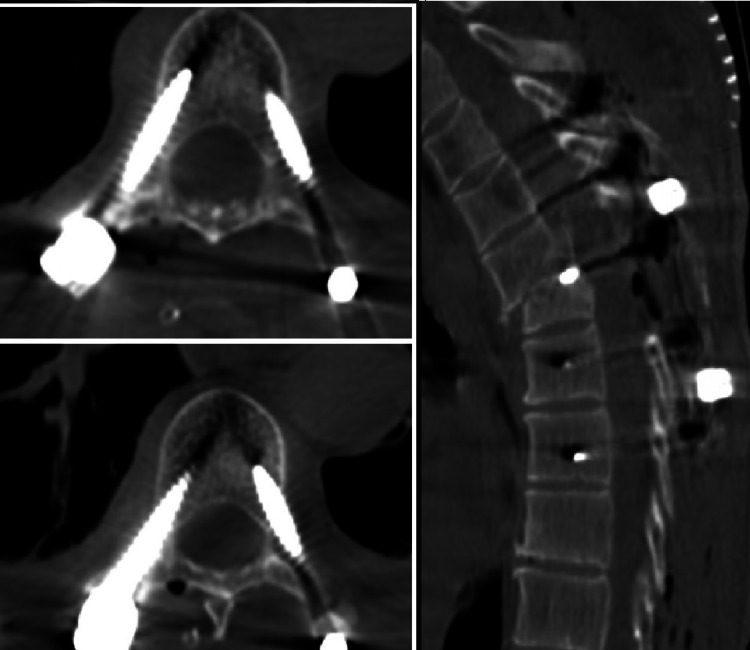
Postoperative CT image of a male patient operated with 3D modeling technique.

The mean TLICS scores for group 1 and group 2 were 6.3 ± 4.2 and 6.7 ± 4.1, respectively. Of the 25 upper thoracic fracture patients (group 1), five (20%) were T3, eight (32%) were T4, and 12 (48%) were T6 fractures, which were operated on by 3D modeling with preoperative planning (Figures 4-6). For these 25 patients, the concordance rate between pedicle positions studied on preoperative models and postoperative pedicle screw positions was 93.8% for T3 fractures, 94.7% for T4 fractures, and 98.4% for T6 fractures (Table 4).

**Table 4:** Compliance of preoperative planning (Group 1) with postoperative pedicle screw positions in the vertebral model.

Vertebral Model with Fracture Diagnosis	Pedicle Screw Size	Number of Pedicle Screws Inserted	Model Integrity Rate
T3	4.0 x 26 mm	24	93.8%
T4	4.0 x 26 mm	57	94.7%
T6	6.0 x 45 mm	93	98.4%

The 3D printing time of vertebral templates produced for each patient was 14 ± 3 minutes. The amount of PLA used for the production of each template was 2 ± 0.42 grams. The total production cost of 25 templates produced for each patient was $5.31.

## Discussion

Upper and middle thoracic fractures are rare among spinal fractures. Approximately 10-20% of general spinal traumas are observed in this region [12]. Upper thoracic vertebral injuries often result in axial stress and bending with rotation and dislocation. This type of injury is generally seen at T4-T6 levels in motorcycle riders [13].

With the development of spine surgery, posterior thoracic interpedicular screwing has become more important. The key to this operation is the correct placement of the pedicle screw in one step, but it has been difficult due to safety concerns relating to the upper thoracic pedicle properties. The upper thoracic spine pedicles are smaller in diameter, and there are different angles for each spine. Thoracic pedicles are short and narrow, and their cortex is thin and fragile; therefore, thoracic pedicles are easily broken during screwing [14]. Additionally, the angles of the thoracic pedicles are different from each other, which has made the rate of error in placing thoracic pedicle screws at one time very high, causing serious consequences by damaging the surrounding tissues [15].

The anatomy of the thoracic pedicles is more complex, and screw insertion is more difficult in complex thoracic fractures and vertebral malformations. The penetration rate can reach 30-40% in the insertion of the thoracic pedicle screw with the freehand technique [16]. CT-based navigation systems are used to guide the placement of pedicle screws on the spine. Its use is not common due to its disadvantages, such as intraoperative position changes and spinal instability, lack of real-time navigation, and high cost [17].

With the use of 3D printing in spine surgeries, the production of guide plates, and provision of preoperative simulation, the accuracy of operations has increased. The fact that the accuracy is not affected by the intraoperative position and the higher reliability of guide plates provides superiority to navigation systems. Providing preoperative simulation and using the model as a guide during surgery reduce the surgeon's margin of error and operation time [18].

Controls performed with 2D fluoroscopy in upper thoracic interpedicular screwing show high error rates. Guzey et al. retrospectively examined 113 pedicle screws between T2-T8 in 24 patients without coronal deformity [19]. The control of the pedicle screws was checked during the operation by C arm fluoroscopy and postoperative CT. The faulty pedicle screw insertion rate was found to be 20.3%, 27.4% between T2-T5 and 14.5% between T6-T8 [19]. Pedicular screws were applied to T4-T12 levels by five experienced surgeons on five fresh cadavers. In postoperative CTs, a faulty screw placement rate was found at a rate of approximately 41%. Of these, 21 screws were observed to be in the vertebral canal by preparing the medial wall of the pedicle [4]. In our study, in patients who underwent surgery using the freehand technique, a total of 28 (16.3%) incorrect pedicular screw placements were observed, 24 screws grade 2 (14%) and four screws grade 3 (2.3%).

With the application of 3D printing in spine surgeries, personalized production of guide plates, and preoperative simulation of the operation on the model have increased the accuracy of operations. Lu et al. used 3D modeling as an aid to cervical pedicle or vertebral plate screw placement and proved that it can provide correct placement of the screws [20]. Customized 3D spine models and screw insertion guide plates can be used to aid screw insertion and ensure the correct insertion of screws.

Mizutani et al. designed 3D models to apply cervical pedicle screws and achieved good results with guide plates in placing cervical pedicle screws [21]. Sugawara et al. created personal 3D navigation models for thoracic pedicle screws and applied pedicle screws under their guidance simply and safely. In 103 patients, 813 screws were placed with 3D guides. In postoperative CT scans, 801 screws (98.5%) were placed without cortical violation, and no injury to the vessels and nerves was observed [9]. Xu et al. placed 56 pedicle screws in seven patients with upper and middle thoracic trauma using the 3D printing-supported preoperative plan method [10]. Regarding the placement of 56 screws according to postoperative CT images, 33 were grade 0, 18 were grade 1, four were grade 2 (perforated sidewall), and one was grade 3 (perforated sidewall, no vascular nerve injury). The accuracy rate was 91% [22]. In our study, screw placement was performed according to postoperative CT images of 174 pedicle screws placed in the upper thoracic spine in 25 patients with preoperative 3D printing support and guidance, and 158 (90.8%) were grade 0, 10 (5.8%) were grade 1, and six (3.4%) were grade 2. Grade 3 positioning was not observed in any screw, and the pedicular screw placement accuracy rate was 96.6%. Comparing the pedicle screw placement accuracy of the upper thoracic vertebrae (96.6%) and the pedicle screw placement accuracy (83.6%) of the freehand technique in the 3D printing-supported group, the difference was statistically significant (p < 0.05).

In the study by Pan et al., 37 patients with spinal deformities were operated on, with group 1 (25 patients, 396 screws) supported by 3D printing and group 2 (25 patients, 312 screws) supported by the freehand method. The operation time in group 1 was 283 ± 22.7 minutes. In group 2, it was 285 ± 25.8 minutes. The operation time was found to be shorter in group 1, although the difference was not statistically significant (p = 0.89) [23]. In our study, whereas the operation time was 134 ± 22 minutes for group 1, it was 152 ± 38 minutes for group 2. The difference in operation times was statistically significant (p < 0.05).

In the study by Clifton et al., for 40 C7, 40 T6, and 40 L5 pedicle screws, the rate of agreement between the pedicle positions studied on preoperative models and the postoperative pedicle screw positions was found to be 100% for C7, 100% for T6, and 93% for L5 [24]. In our study, five (20%) of the 25 upper thoracic fracture patients (group 1) were T3, eight (32%) were T4, and 12 (48%) were T6 fractures, which were operated on by preoperative planning using 3D modeling. For these 25 patients, the concordance rate between pedicle positions studied on preoperative models and postoperative pedicle screw positions was 93.8% for T3 fractures, 94.7% for T4 fractures, and 98.4% for T6 fractures.

Vertebral screw misplacement and vascular injuries are common in the upper thoracic region [25]. The ability to perform preoperative surgical simulation of the 3D printing-supported model, the application of the upper thoracic pedicle screw will become more efficient and easier. In our study, the accuracy rate obtained in the 3D printing-supported group was 96.5%, which was higher than that of the freehand technique group. We think that the 3D printing-supported method in upper thoracic pedicle screw application will shorten learning time, provide easier learning on the model, and increase pedicular screw placement accuracy.

## Conclusions

For upper thoracic pedicle screw insertion 3D models, guide plates can be produced inexpensively and individually. It provides a new method for accurate placement of upper thoracic pedicle screws with high accuracy and comfortable use in screw insertion.
